# The causes and frequency of kidney allograft failure in a low-resource setting: observational data from Iraqi Kurdistan

**DOI:** 10.1186/s12882-021-02486-9

**Published:** 2021-08-07

**Authors:** Alaa Abbas Ali, Safaa E. Almukhtar, Kais H. Abd, Zana Sidiq M. Saleem, Dana A. Sharif, Michael D. Hughson

**Affiliations:** 1 University of Sulaimani College of Medicine, Quirga Road, Sulaimani, Iraq; 2 University of Hawler College of Medicine, Erbil, Iraq; 3 University of Dohuk College of Medicine, Dohuk, Iraq

**Keywords:** Kidney transplantation, Kidney allograft pathology, Allograft rejection, Acute and chronic allograft failure

## Abstract

**Background:**

In the developing world, transplantation is the most common long-term treatment for patients with end-stage renal disease, but rates and causes of graft failure are uncertain.

**Methods:**

This was a retrospective outcomes study of renal transplant patients seen in Iraqi Kurdistan nephrology clinics in the year 2019. In 2019, 871 renal transplant patients were registered and outcomes followed through 12/31/2020. Indicated renal biopsies were obtained on 431 patients at 1 day to 18 years post-transplantation. Outcomes were compared with United States Renal Data System (USRDS) living donor reports.

**Results:**

All donors were living. The recipient age was 38.5 ± 13.3 years, 98.2% were < 65 years old, 3.7% had previous transplants, and 2.8% had pretransplant donor-specific antibodies (DSA). Gehan-Breslow estimated failure rates for all-cause, return to HD, and death with functional graft were 6.0, 4.2, and 1.9% at 1 year and 18.1, 13.7, and 5.1% at 5 years post-engraftment (USRDS 2000; 1 year: 7.0, 5.0, 2.6%; 5 year: 22.3, 15.2, 10.6%. USRDS 2010; 1 year: 3.7, 2.4, 1.4%; 5 year: 15.3, 9.6, 7.3%). The median graft survival was 15 years. Acute tubular injury (ATI), infarction, and acute T cell-mediated rejection accounted for 22.2% of graft loss, with > 75% of these failures taking place in the first year. Most graft failures occurred late, at a median post-transplant time of 1125 (interquartile range, 365–2555) days, and consisted of interstitial fibrosis and tubular atrophy (IF/TA) (23.8%), transplant glomerulopathy (13.7%), and acquired active antibody-mediated rejection (12.0%). The significant predictors of graft loss were C4d + biopsies (*P* < 0.01) and advanced IF/TA (*P* < 0.001).

**Conclusions:**

Kurdistan transplant patients had graft failure rates similar to living donors reported by the USRDS for the year 2000 but higher than reported for 2010. Compared to USRDS 2010, Kurdistan patients had a moderate excess of HD failures at one and 5 years post-engraftment. Nevertheless, prolonged survival is the norm, with chronic disorders and acquired DSA being the leading causes of graft loss.

## Introduction

An estimated 80% of the worlds’ noncommunicable diseases, including chronic kidney disease (CKD), is found in low and middle-income countries [1]. As CKD progresses to end-stage renal disease (ESRD), developing nations face an increased burden of expensive care. Because of the cost and complexity of hemodialysis, kidney transplantation is the most frequently used maintenance therapy for ESRD in most of the world [2, 3]. Historically, high rates of acute rejection and the cardiovascular and infectious complications of corticosteroid immunosuppression were the principal obstacles to kidney transplantation. Over the past four decades, calcineurin inhibitors (CNI) have decreased the frequency and severity of T cell-mediated rejection (TCMR), and patients can expect a reasonably normal lifestyle, at least in the short-term [2, 3]. In most of the Middle and Near East, the standard practice model screens for pre-existing donor-specific antibodies (DSA) and selects mainly non-sensitized patients for transplantation [4, 5]. Nephrologists accept chronic allograft changes as generic complications of engraftment and only recently have recognized the importance of de novo antibody-mediated rejection (AMR).

CNI-based immunosuppressive regimens have produced a notable improvement in one- and five-year graft survival, but similar gains at 10 years and beyond are limited [6–8]. While cell-mediated rejection is mostly preventable and treatable, T cell therapies have little influence on AMR, and AMR is becoming a frequent cause of late graft loss [9, 10].

In the modern era of immunosuppression, investigations into the causes of transplant failure were initiated by El Zoghby et al. [7]. These authors identified transplant glomerulopathy (TG) as a common cause of late graft failure and considered TG an alloimmune disorder. Subsequently, The recognition of C4d + and C4d- forms of AMR lead to the 2017 revision of the Banff Classification of Renal Allograft Pathology [10–12]. This classification provides criteria for diagnosing acute and chronic AMR and regards TG as a C4d- antibody disease [10–12].

Renal transplantation in Iraq is in transition. Until 2003, Baghdad was its center. War then forced many surgeons and nephrologists into the north’s Kurdish region or out of the country [13, 14]. Most transplant centers have pretransplant antibody and HLA testing, but continuous supplies of reagents and expertise in the interpretation of results present challenges. The central government supports the cost of immunosuppressive therapy, yet financial shortfalls are a constant problem that shifts costs periodically onto the patient and strains compliance. These may be considered deficiencies in transplant practice, but they are common in most developing countries. CNI-based immunotherapy has certainly reduced acute TCMR, but the remaining causes and outcomes of graft dysfunction are not generally appreciated [4, 5].

In our regional biopsy practice, acute transplant disorders are commonly seen early after transplantation, but chronic transplant changes comprise more than half of our diagnoses. To evaluate chronic, as well as, acute antibody-mediated transplant rejection, routine C4d staining was started on all transplant biopsies in 2018. The current study investigates the frequency of the 2017 Banff categories of transplant diagnoses and outcomes in our middle-income but technically developing country. We are attempting to determine: 1) our regional rates of graft failure, and 2) how the causes of graft failure are distributed among different diagnoses.

## Material and methods

The study was observational for a defined period of time. The STROBE reporting checklist for cross-sectional studies was followed [15]. The inclusion criteria consisted of registration in one of the three Kurdistan nephrology services in the year 2019 and follow-up through the end of 2020. The exclusion criteria consisted of a patient not being a nephrology clinic registrant in the year 2019. The study additionally involved the evaluation of transplant kidney biopsies performed on the 2019 registrants. Inclusion criteria for biopsies were nephrology clinic registration in 2019. Exclusion criteria for biopsies consisted of the patient not being a nephrology clinic registrant in 2019.

### Patient and biopsy sample selection

The nephrology services of the Kurdish region of Iraq are located in the cities of Dohuk, Erbil (Hawler), and Sulaimania. The centers serve approximately 5,200,000 residents of Kurdistan and a large number of patients from southern Iraq, primarily from Baghdad. From January 1 to December 31, 2019, the services registered 871 renal transplant patients, 183 in Dohuk, 298 in Sulaimania, and 390 in Erbil. Of the 871 patients, 542 (62.2%) were transplanted in 2019.

Renal biopsies are centralized in Sulaimania. In 2019, the renal biopsy laboratory evaluated satisfactory kidney allograft biopsies from 301 of the 871 patients. For the 2019 biopsies, the median post-transplant time was 150 (IQR 30 to 940) days. In 2020, an additional 130 of the 871 patients had biopsies. This provided biopsies from a total of 431 patients who were clinic registrants in 2019. The 2020 biopsies had a median post-transplant time of 1095 (IQR 450 to 2464) days and, by the exclusion criteria, did not include any specimens from patients transplanted after December 31, 2019.

Biopsies were performed for the clinical indications of unexpectedly low (primary) function post-transplantation (14.1%), deterioration of graft function from a previous baseline level (77.9%), and proteinuria (7.9%). Recorded patient data consisted of age, gender, history of diabetes, time post-transplantation, serum creatinine (Scr), proteinuria, immunosuppressive regimen, source of the donor, and the presence or absence of DSA.

Preemptive transplantation comprised approximately 15% of procedures. For most patients, dialysis was between 2 to 4 months, and for all patients, dialysis was less than 1 year. The standard dialysis schedules throughout Iraq are 4 hours sessions, twice a week. All dialysis is in-center hemodialysis.

All transplants were ABO compatible. Luminex™ (Austin, TX) microbead assays and Immucor™ (Norcross, GA) kits were used to screen patients for donor-specific antibodies (DSA) and type patients and donors for 51 HLA-A, HLA-B, and HLA-DR antigens. Virtual cross-matching was performed prior to transplantation. Patients with pretransplant DSA were treated with ATG, plasmapheresis, and intravenous immunoglobulin and transplanted when the mean fluorescent intensity of the DSA became undetectable.

The most frequent combination of immunosuppressive therapy was tacrolimus (0.075–0.14 mg/kg/day) or cyclosporin (4–6 mg/day), mycophenolate mofetil (2 g/day), and methylprednisolone (initially 1 mg/kg/day, tapered to 5 mg/day). Episodes of acute TCMR were treated with methylprednisolone (7.5 mg/kg) and, if not responsive, with anti-thymocyte globulin (ATG) at 1–2 mg/kg/dose or higher depending upon the response. T cell depletion with ATG was routinely provided at the time of transplantation.

Whole blood tacrolimus or cyclosporin was measure twice during the first week post-transplantation, every 2 weeks during the third to sixth months, every 2 months from 6 months to 1 year, and every 4 to 6 months after 1 year. The target trough levels of tacrolimus are 10–15 ng/ml until the third month, 7–10 ng/ml until 1 year, and 5–7 ng/ml thereafter. Target cyclosporine levels are 500–600 ng/ml until the third month, 400–600 ng/ml until 1 year, and 350–400 ng/ml afterward. Patients are additionally tested if there is clinical suspicion or a biopsy diagnosis of CNI toxicity or rejection.

### Biopsy preparation and criteria for diagnoses

Biopsies were studied by light microscopy in serial sections using hematoxylin and eosin, periodic acid –Shiff (PAS), Masson trichrome, and Jones periodic acid-methenamine silver stains. Electron microscopy was not performed on any biopsy. Direct immunofluorescence (IF) was performed on frozen sections using fluorescein-conjugated anti-human IgG, IgM, IgA, C3, and C1q (DAKO, Santa Clara, CA). C4d staining was performed by indirect IF on frozen sections using a monoclonal anti-C4d antibody (Bio-Rad, Inc).

Diagnoses and the semiquantitative scoring of histologic lesions were based on the Banff 2017 Classification of Renal Allograft Pathology [11]. The diagnoses were categorized into acute tubular injury (ATI), acute TCMR, chronic active TCMR, active AMR, interstitial fibrosis and tubular atrophy (IF/TA), CNI toxicity, recurrent or de novo kidney disease (KD), BK nephropathy, and acute pyelonephritis. Acute TCMR included borderline rejection. Active AMR was defined by the presence of C4d deposition in peritubular capillaries and was subdivided into hyperacute rejection, active AMR, mixed acute TCMR with active AMR, and chronic active AMR. We placed biopsies in the category of transplant glomerulopathy (TG) when they demonstrated double contour glomerular basement membranes (GBM) in the absence of C4d peritubular capillary staining after excluding thrombotic microangiopathy or glomerulonephritis as reasons for the glomerular changes [11]. Acute tubular necrosis, non-specific histological changes, and histologically normal biopsies for patients with diminished graft function were classified as ATI. Thirty-nine patients had more than one biopsy, with the earliest biopsy being used for the diagnosis.

### Method of patient follow-up

**Fig. 1:**
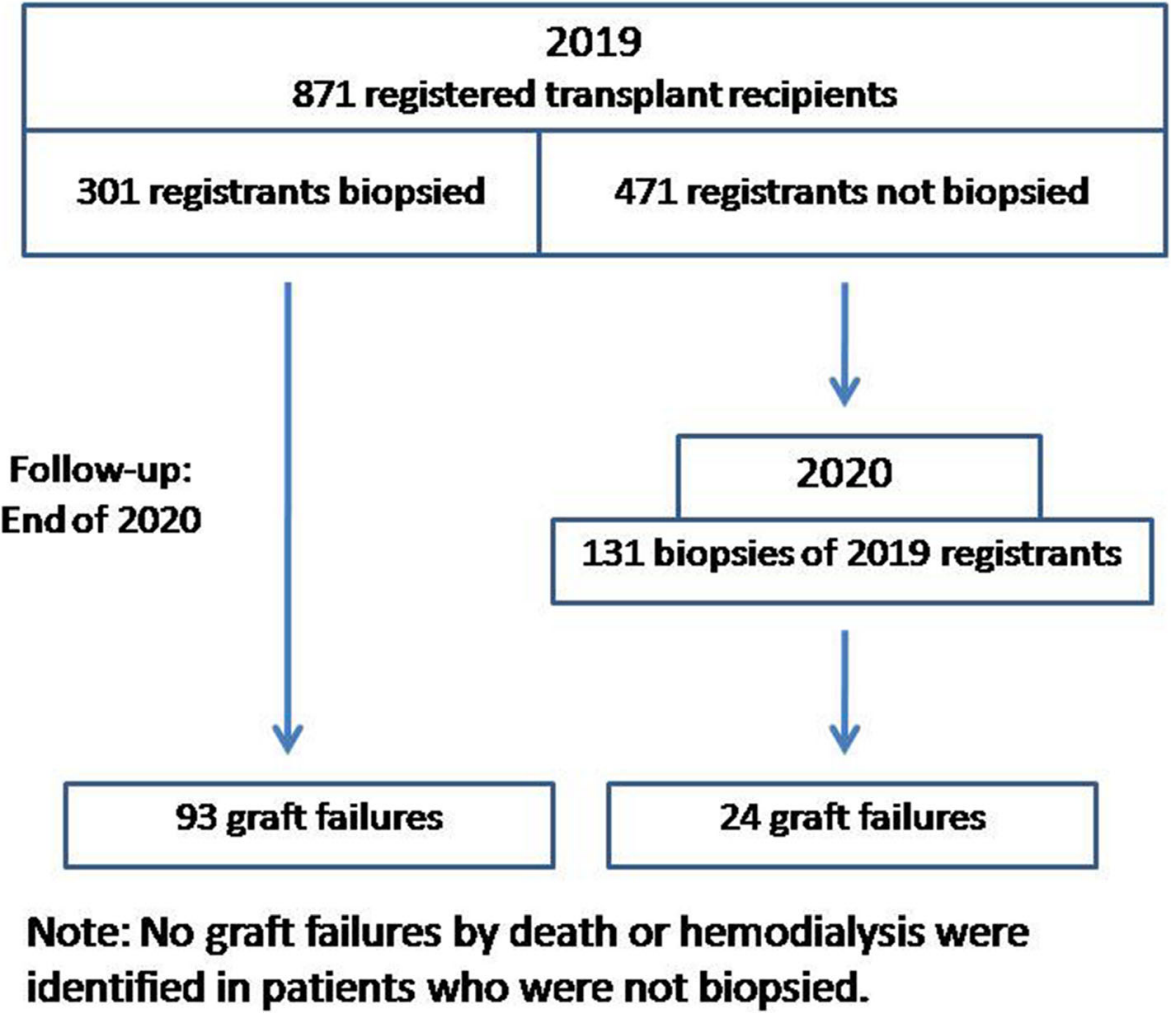
Flowchart showing the patient population consisting of registrants in Iraqi Kurdistan transplant clinics in the year 2019 and followed-up through the end of 2020. The number of biopsies and the number of graft failures are indicated

Patient follow-up was conducted at the end of 2020. Follow-up procedures consisted of patient or family telephone contact or a nephrologist’s review of clinical notes and hospital records. Thirty-nine of the 871 patients did not return to the clinic or could not be contacted (4.5%) and were lost to follow-up. The average follow-up period was 18.6 ± 3.3 months, with the follow-up ending on December 31, 2020. Outcomes were designated as graft failures requiring hemodialysis (HD), death with a functioning graft (DWFG), and functioning graft. Deaths while on dialysis were considered HD failures. Figure 1 provides a flowchart of the patient follow-up with the number of biopsied patients and the number of graft failures.

### Statistical procedures

Data were entered into Microsoft Excel 2007 (Redmond, WA) worksheets and analyzed with IBM SPSS 26 (Armonk, NY), Excel, and SigmaStat version 3.5 (San Jose, CA) software. Differences for age, time post-transplantation, and serum creatinine were tested as multiple group comparisons by a Kruskal-Wallis analysis of variance on ranks. Graft failure estimates used Kaplan Meier (Gehan-Breslow) functions. The time of failure was the time post-transplantation recorded at biopsy. For censored patients, the follow-up period was the post-transplant time plus the average follow-up time of 18.6 months (558 days). All survival consisted of DWFG and HD. Death-censored outcomes consisted of HD. Proportional differences between discrete variables were analyzed by Chi-square or Fisher Exact tests. Spearman correlations evaluated the relationships between the time of transplantation and scores for IF/TA and TG. Reverse step-wise logistic regression tested the significance of the independent variables of age, sex, Kurdish/Arab ethnicity, donor source, IF/TA, C4d positive IF, AKI, and acute TCMR to the outcomes of HD or DWFG, with variables eliminated if *P* ≥ 0.10. Differences were considered significant at *P* < 0.05.

## Results

### Patient demographics

The characteristics of the 871 patients are summarized in Table 1. The average patient was 38.5 ± 13.3 years old, with 19.5% being ≥50 years old and 1.8% ≥ 65 years old. Recipients were 77.3% male; 6.5% were diabetic. All donors were living; 16.2% were related, with only five spousal (wife to husband) donations. Ethnicity was determined in biopsied patients by the father’s name on the requisition forms. Of the 431 biopsied patients, 233 (54.1%) were Kurdish. Pre-transplant donor-specific antibodies (DSA) were found in 2.8% of patients; 3.7% of patients were previously transplanted. Patients were biopsied from 1 day to 18 years post-transplant. The median time for biopsy post-transplantation was 365 (IQR 52–1290) days.

**Table 1:** Characteristics of transplant recipients

Characteristic	Value n (%)
Number of patients	871
Average age, years	38.5 ± 13.3
> 50 years old	169 (19.5)
Males	673 (77.3)
Diabetes	58 (6.5)
Living donor (compensated)	719 (82.4)
Living donor (spouse)	5 (0.6)
Related donor (non-spouse)	141 (16.2)
Pre-emptive (estimated)	(15)
Time of biopsy post-transplantation, days	365, range 1 to 6570 (IQR, 52–1290)
Ethnic Kurd^a^	233 (54.1)
Previous transplant^a^	16 (3.7)
Pretransplant DSA positive^a^	12 (2.8)
Stopped immunosuppression ^a^	28 (6.5)
Maintenance immunosuppression^a^	
CNI (tacrolimus/cyclosporine), MMF, steroid	268 (62.1)
CNI (tacrolimus/cyclosporine), steroid	163 (37.9)
Indication for biopsy	
Primary poor function	61 (14.1)
Deterioration of graft function	336 (77.9)
Proteinuria	34 (7.9)

Abbreviations: Time of biopsy is expressed as median and interquartile range (IQR)

*CNI* calcineurin inhibitor, *MMF* mycophenolate mofetil, *DSA* Donor-specific antibody

^a^ Data available for biopsied patients only

For biopsied patients, submission forms indicated that 361 patients (83.6%) were taking immunosuppressive medication and that 28 patients (6.5%) had stopped. Medication use was not stated for 43 patients (10.0%).

### Pathology results

Table 2 shows the results for 431 biopsies that include 301 biopsies obtained in 2019 and 130 in 2020. The most common diagnosis was acute TCMR at 25.5%, followed by AKI at 15.1%, and IF/TA at 13.2%. The next most frequent diagnosis was active C4d + AMR in 9.7% of patients. Active AMR included hyperacute rejection (2 patients), active AMR (11 patients), chronic active C4d + antibody rejection (21 patients), and mixed acute TCMR and active AMR (8 patients). Two of the 11 patients with active AMR and one with mixed cellular and AMR had DSA detected before biopsy that was not identified before transplantation. None of the patients with chronic active AMR were tested for DSA before or after biopsy.

**Table 2:** Pathological diagnoses of 431 transplant biopsies. The median (interquartile range) post-transplant time and average serum creatinine (Scr) are provided for each diagnosis

Diagnoses	n (%)	Post-transplant, days	Scr (mg/dL)
ATI	65 (15.1)	40 (14–120)	2.1 ± 0.9
Infarction	9 (2.1)	14 (7–30)	3.9 ± 1.9*
Acute TCMR	110 (25.5)	135 (24–365)	2.3 ± 1.1
AMR C4d+			
Hyperacute	2 (0.4)	1,3	5,7
active AMR	11 (2.6)	20 (11–27)	2.9 ± 2.1
Chronic active AMR	21 (4.9)	2190 (1460–2555)	2.5 ± 1.1
Acute TCMR and AMR	8 (1.9)	52 (14–431)	3.2 ± 2.0
Chronic TCMR	22 (5.1)	923 (425–1734)	3.1 ± 2.2
TG	38 (8.8)	2373 (1140–2920)	2.5 ± 1.1
IF/TA	57 (13.2)	1153 (520–2920)	2.5 ± 1.3
Recurrent or de novo KD	37 (8.6)	1095 (650–1460)	2.2 ± 1.2
CNI toxicity	24 (5.6)	195 (64–1095)	2.0 ± 0.7
Acute pyelonephritis	14 (3.2)	305 (75–913)	2.7 ± 1.2
BK virus nephropathy	11 (2.6)	340 (180–548)	2.2 ± 0.7
Incidental^a^	2 (0.01)	15, 180	1.7, 1.9
All diagnoses	431 (100)		ANOVA *P* = 0.01^b^

*Abbreviations*: *TCMR* T cell-mediated rejection, *AMR* antibody-mediated rejection, *TG* transplant glomerulopathy, *IF/TA* interstitial fibrosis/tubular atrophy, *ATI* acute  tubular injury, *CNI* calcineurin inhibitor, *KD* kidney disease. ^a^ Incidental: Dihydroxyadenine crystal nephropathy, karyomegalic interstitial nephritis. ^b^ Scr levels for infarction are significantly different than other diagnostic groups. There is no significant difference in Scr between diagnoses other than infarction

**Fig. 2:**
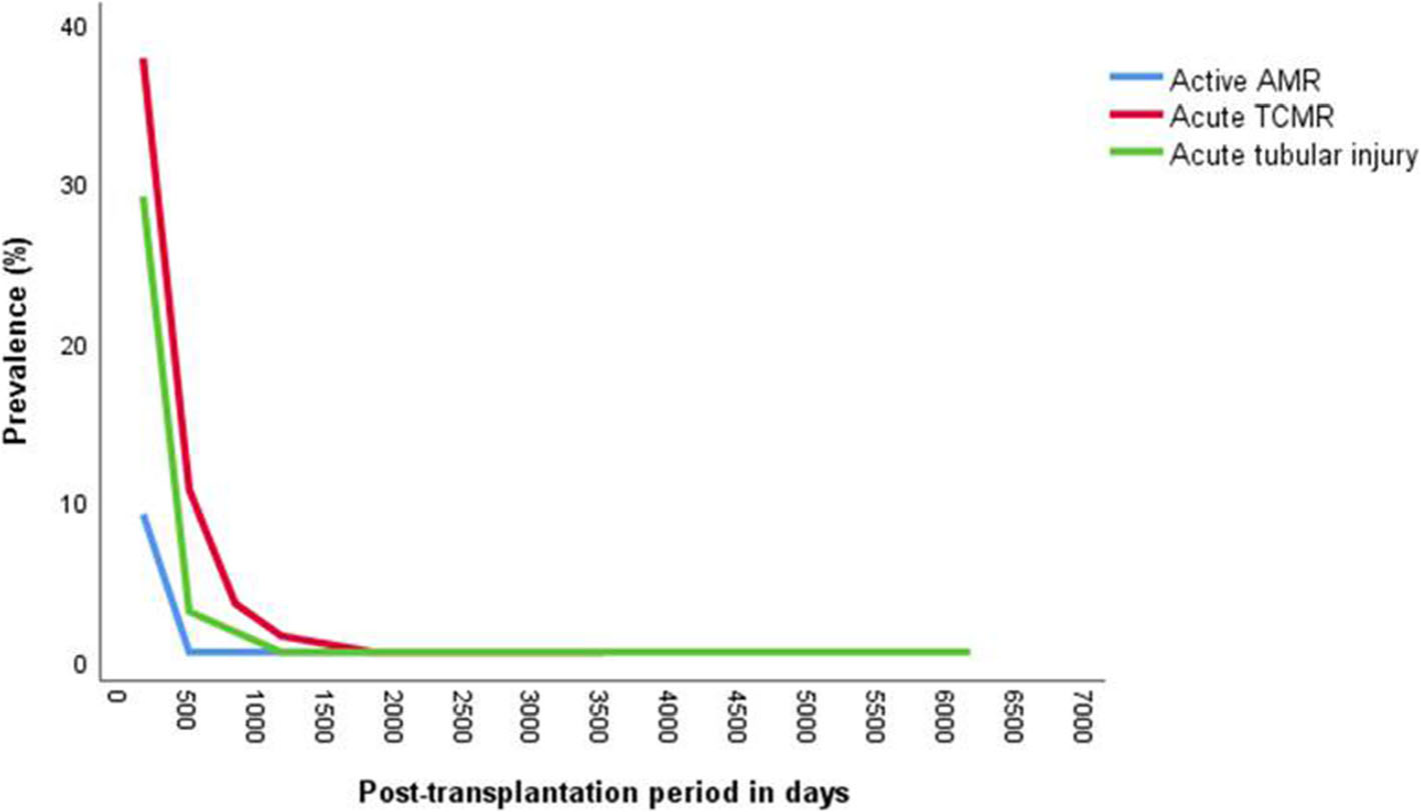
Distribution of acute transplant diagnoses by time post-transplantation (431 biopsies). Legend: Acute tubular injury and acute TCMR were primarily first year changes, with rare late occurrences. Active AMR was seen with pre-transplant and acquired DSA. Abbreviations: TCMR, T cell-mediated rejection; AMR, antibody-mediated rejection; DSA, donor specific antibodies

The distribution of cases of ATI, acute TCMR, and active AMR are illustrated in Fig. 2. Most patients with ATI and all patients with infarction were found early after implantation. Twenty-six percent of ATI was seen after 100 days and 12% after 12 months. These later AKTI biopsies did not show features of CNI toxicity or infection, and the cause was unknown.

The median time of biopsy for acute TCMR was 135 days post-transplantation, but with 25 cases (23%) being diagnosed sporadically at one to 10 years. Acute TCMR tended to be mild, with 76% being classified as grade I tubulointerstitial disease and 26% as grade II cellular rejection with vascular involvement.

Seven acute TCMR patients had stopped taking immunosuppressive medication, but 71 reported using medications as prescribed. The two groups showed no significant differences in the histologic scores of inflammation (*P* = 0.12), tubulitis (*P* = 0.59), or vasculitis (*p* = 0.48), and there was no difference in their rates of graft failure (stopped medication, one failure; using medication 11 failures, *P* = 0.17).

**Fig. 3:**
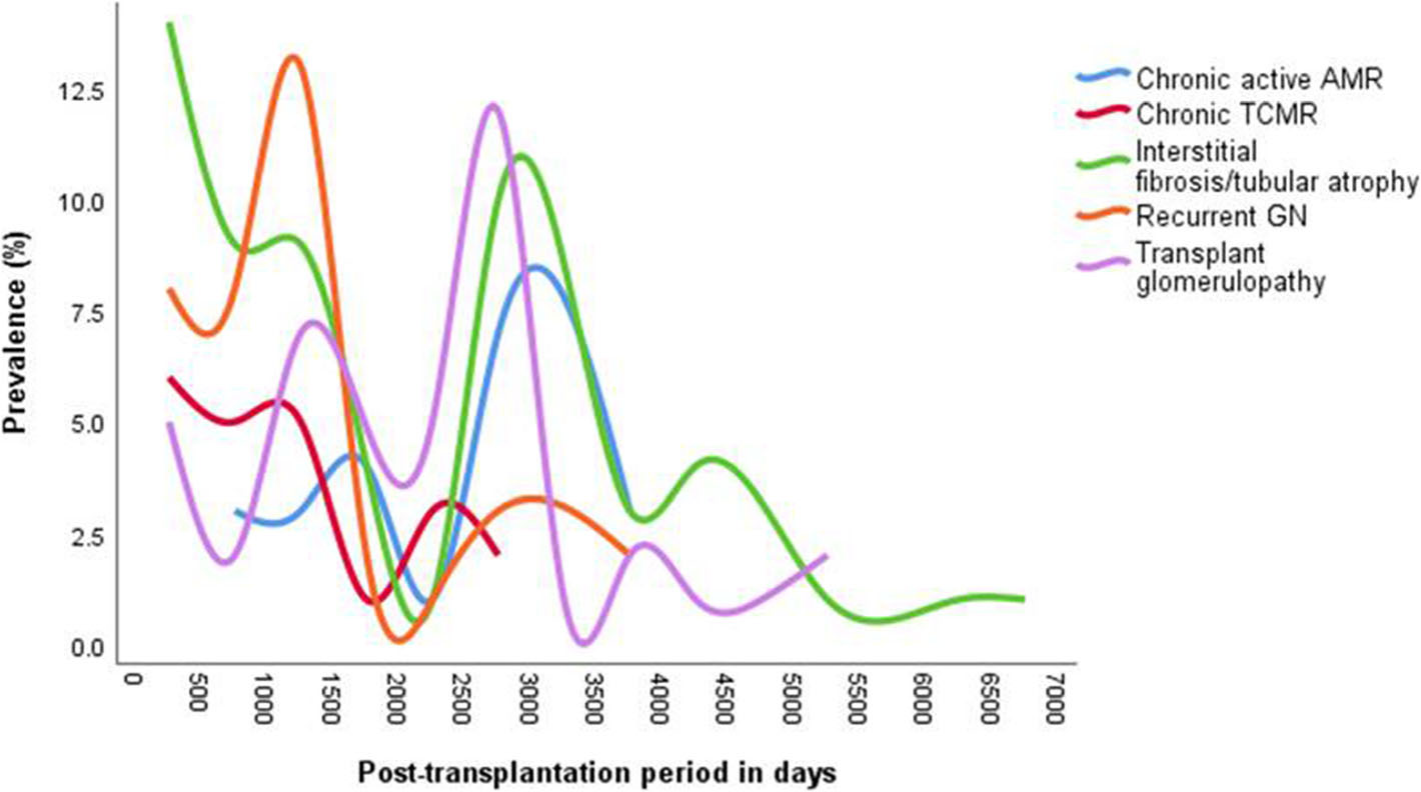
Distribution of chronic transplant diagnoses by time post-transplantation (431 biopsies).Legend: Transplant glomerulopathy (TG) and interstitial fibrosis/tubular atrophy were the most frequent causes of late graft dysfunction and were found at the end of the first year until 18 years post-transplantation. Chronic active AMR paralleled TG. Abbreviations: AMR, antibody-mediated rejection; TCMR, T cell-mediated rejection; GN, glomerulonephritis, mainly FSGS

TG and IF/TA were found mainly after 2 years and rarely late in the first year post-transplantation. Figure 3 compares the frequency of diagnosis of chronic active AMR, TG, IF/TA, chronic TCMR, and recurrent or de novo KD. The most common chronic conditions were IF/TA in 57 and TG in 38 patients. Chronic active AMR was not distinguishable from TG by histology, time of occurrence, or Scr levels. The difference was the interstitial capillary C4d staining in chronic active AMR that was, by definition, negative in TG. The ratio of chronic active AMR to TG was 1:1.8 (56%).

Thirty-seven patients had recurrent or de novo KD, with 18 diagnosed as FSGS. All FSGS occurred after the first year over a range of 520 to 3650 days. Five de novo KD patients relapsed in the first 300 days, two with membranous glomerulonephritis and three with a TMA.

Twenty-four patients were diagnosed with CNI toxicity. In the first year, CNI toxicity consisted of a TMA (*n* = 11) or isometric tubular vacuolization (*n* = 2). After 1 year (*n* = 11), CNI toxicity consisted of IF/TA with nodular arteriolar hyalinosis and sometimes segmental glomerulosclerosis.

There was a somewhat different distribution of diagnoses in 2019 and 2020 (Table 3). This was because biopsies were not performed on patients transplanted after December 31, 2019. This excluded post-transplant AKTI in 2020 and included only patients with clinically silent disease in 2019 that were found to have proteinuria and/or increased serum creatinine in 2020. There was a significantly increased proportion of chronic active AMR in 2020 that we cannot explain.

**Table 3:** Distribution of biospy diagnosies in 2019 and 2020

Diagnoses	2019 n (%)	2020 n (%)	*P* (year)
ATI	58 (19.3)	7 (5.4)	0.01
Infarction	9 (3.0)	0	–
Acute TCMR	78 (25.9)	32 (24.6)	0.92
AMR C4d+			
Hyperacute	2 (0.4)	0	–
Active AMR	10 (3.3)	1 (0.1)	0.24
Chronic active AMR	7 (2.3)	14 (10.8)	0.001
Acute TCMR and AMR	7 (2.3)	1 (0.1)	0.49
Chronic TCMR	11 (3.7)	11 (8.5)	0.09
TG	23 (7.6)	15 (11.5)	0.31
IF/TA	36 (12.0)	21 (16.2)	0.38
Recurrent or de novo KD	23 (7.6)	14 (10.8)	0.43
CNI toxicity	19 (6.3)	5 (3.8)	0.46
Acute pyelonephritis	8 (2.7)	6 (4.6)	0.47
BK virus nephropathy	8 (2.7)	3 (2.3)	0.90
Incidental^a^	2 (0.01)	0	–
All diagnoses	301	130	
Time post-transplant (days)	120 (23–843)	1095 (450–2190)	< 0.001

*Abbreviations*: *TCMR* T cell-mediated rejection, *AMR* antibody-mediated rejection, *TG* transplant glomerulopathy, *IF/TA* interstitial fibrosis/tubular atrophy, *ATI* acute tubular injury, *CNI* calcineurin inhibitor, *KD* kidney disease. ^a^ Incidental: Dihydroxyadenine crystal nephropathy, karyomegalic interstitial nephritis. The time post-transplant is expressed as median (interquartile range)

### Allograft failures

By the end of 2020, graft failure occurred in 117 patients (Table 4). In the first year post-transplant, 49 grafts were lost, with the most frequent diagnoses being ATI, *n* = 8; infarction, *n* = 7; and acute TCMR, *n* = 11. After receiving second transplants, two patients with pre-existing DSA lost grafts within 7 days because of hyperacute rejection.

**Table 4:** Graft failures by diagnosis, age, hemodialysis failure, and post-transplant time to graft loss

Diagnosis	n (%) all-cause	n (%) HD	Patient age years	Post-transplant Time, days
ATI	8 (6.8)	4 (4.5)	42.0 ± 11.7	16 (7–30)
Infarction	7 (6.0)	5 (5.7)	31.4 ± 13.1	14 (7–22)
Acute TCMR	11 (9.4)	7 (8.0)	39.6 ± 14.2	120 (12–333)
AMR C4d+				
Hyperacute	2 (1.7)	2 (2.3)	35,35	1,3
Active AMR	4 (3.4)	3 (3.4)	33 ± 15.2	16 (9–32)
Chronic active AMR	9 (7.7)	9 (10.2)	44.8 ± 10.9	2555 (2190–2555)
Acute TCMR and AMR	2 (1.7)	2 (2.3)	29,50	14, 1825
Chronic TCMR	8 (6.8)	7 (8.0)	37.8 ± 10.0	1245 (1003–1909)
TG	16 (13.7)	13 (14.8)	42.3 ± 12.6	2372 (1027–2646)
IF/TA	28 (23.8)	22 (25.0)	37.9 ± 14.4	1552 (548–3102)
Recurrent or de novo KD	11 (9.4)	6 (6.8)	37.7 ± 13.5	810 (360–1825)
CNI toxicity	3 (2.6)	3 (3.4)	57.3 ± 15.5	910 (560–1185)
pyelonephritis	3 (2.6)	2 (2.3)	44.7 ± 18.1	1095 (593–1368)
BK virus nephropathy	5 (4.3)	3 (3.4)	56 ± 11.7	365 (340–740)
	117	88 (75.2)		

Values for age and transplant time are for all-cause failure. Age is expressed as mean ± SD and post-transplant time as median (interquartile range). *Abbreviations*: *HD* hemodialysis, *DWFG* death with functional graft, *ATI* acute  tubular injury, *TCMR* T cell-mediated rejection, *AMR* antibody-mediated rejection, *TG* Transplant glomerulopathy, *IF/TA* interstitial fibrosis and tubular atrophy, *CNI* calcineurin inhibitor, *KD* kidney disease. The ages between the diagnostic groups are not statistically different, ANOVA, *P* = 0.084

Infarction, representing 6% of graft loss, was attributed to defective vascular anastomoses. Graft loss with ATI presented a complicated picture. Four of eight failures were DWFG. One patient had graft biopsies showing ATI on days 90 and 120 and then IF/TA grade III requiring HD at 455 days. Another patient with ATI and grade I IF/TA on day 1095 progressed to IF/TA grade III and HD at 1460 days. Because the first diagnosis in these cases was ATI, ATI was the assigned cause of failure.

The pathology of graft loss because of acute TCMR was similarly complicated. Three of the 11 patients had vasculitis. One patient had a Banff grade IIA acute TCMR on day six that progressed to a TMA with cortical infarction and HD on day 66 post-transplantation. Another patient had a Banff grade IB acute TCMR at 365 days that advanced to IF/TA grade III and HD by day 1135. Four of the 11 acute TCMR failures were DWFG.

After the first post-transplant year, 68 patients lost grafts (Table 5). The most common diagnoses were IF/TA, *n* = 28; TG, *n* = 16; chronic active AMR, *n* = 14; and recurrent or de novo KD, *n* = 11. DWFG was responsible for 24.8% of graft loss and occurred nearly equally in the first post-transplant year (*n* = 15) and afterward (*n* = 14). The attributed cause of 13 cases of DWFG was infection, with the infections including four COVID-19 cases. Four patients over 50 years of age had sudden deaths that may have been myocardial.

**Table 5:** Death with a functional graft by biopsy diagnosis, number of deaths, age, post-transplant time, and causes of death

Biopsy diagnosis	n	Age, years	Post-transplant time days	Causes of death
ATI	4	41 (37–46)	15 (2–108)	Infection (2), COVID-19, unk
Infarction	2	26,53	7,42	Infection (2)
aTCMR	4	39 (33–40)	110 (9–232)	Infection, unk (3)
aAMR	1	54	22	Myocardial?
cTCMR	1	47	730	Unk
TG	3	48 (43–58)	1460 (958–2007)	COVID-19 (3)
IF/TA	6	50 (28–53)	970 (609–1695)	COVID-19, PTLPD, infection, myocardial?, unk (2)
Recurrent/denovo KD	5	38 (36–41)	420 (240–2190)	Myeloma, AA amyloidosis, pancreatitis, myocardial?, unk
Pyelonephritis	1	52 (38–55)	1095 (593–1368)	Infection
BK virus nephropathy	2	58 (57–64)	365 (340–740)	Infection, myocardial?
	29			

Values for age and post-transplant time are expressed as median (interquartile range)

*Abbreviations*: *HD* hemodialysis, *ATI* acute  tubular injury, *aTCMR* acute T cell-mediated rejection, *aAMR* active antibody-mediated rejection, *cTCMR* chronic T cell-mediated rejection, *TG* Transplant glomerulopathy, *IF/TA* interstitial fibrosis and tubular atrophy, *CNI* calcineurin inhibitor, *KD* kidney disease, *unk* unknown, *PTLD* post-transplant lymphoproliferative disease

**Fig. 4:**
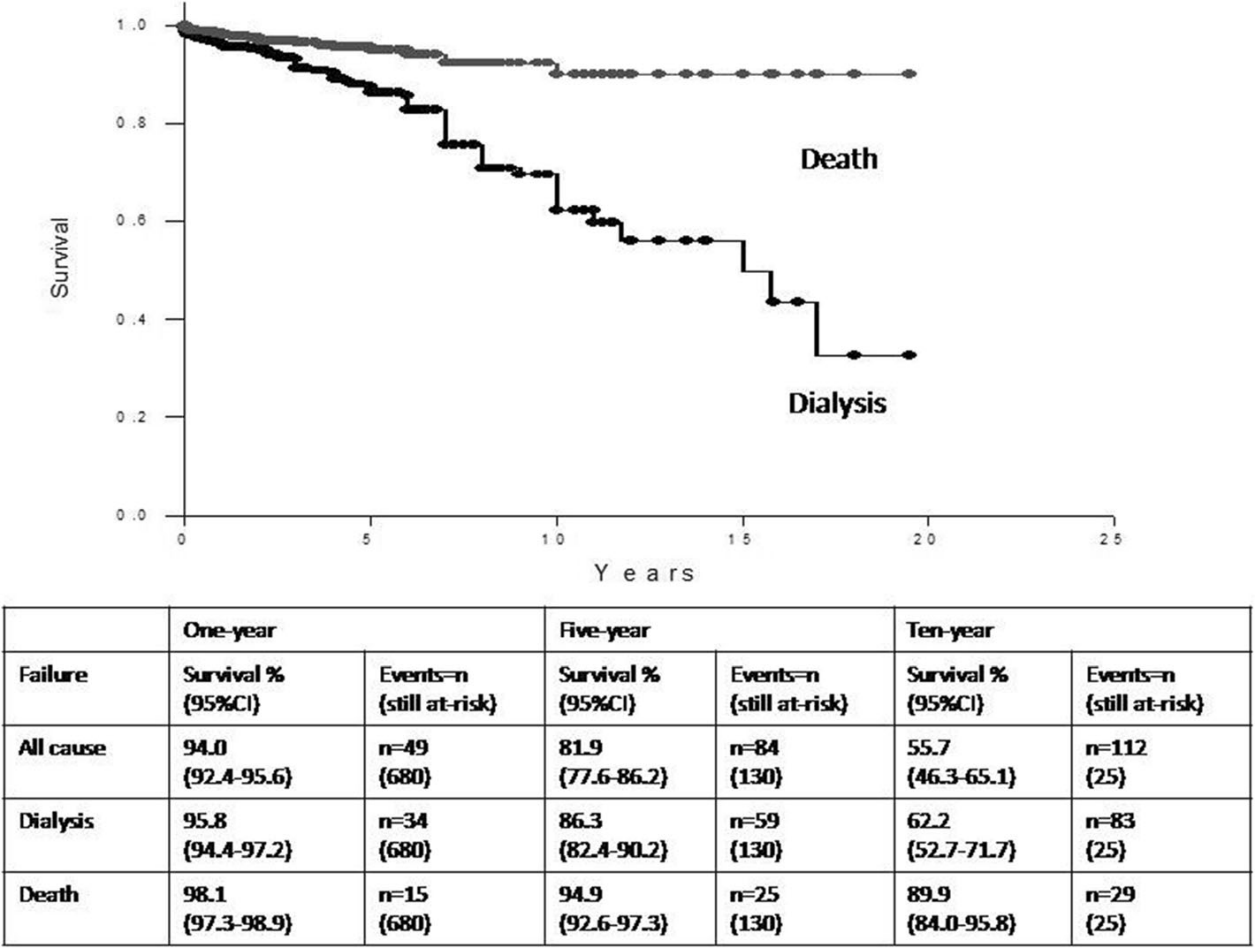
Kaplan-Meier (Breslow) survival by death and dialysis among 871 Kurdistan transplant patients. Legend: Events (n) are the number of cumulative failures over the indicated time interval. Still at-risk are the number of patients remaining after the indicated time interval

Estimated Iraqi Kurdistan one-, five-, and 10-year survival rates for HD and death are shown graphically in Fig. 4, with all-cause survival being added to the underlying chart. The all-cause survival rate was 94.0% at 1 year. The all-cause rate declined to 81.9% at five and 55.7% at 10 years, with a 10-year death censored survival rate of 66.2%.

Because the follow-up period was limited to an average of 18.6 months, the number of patients at-risk fell quickly after 1.6 years and left only 130 patients after 5 years and 25 patients after 10 years. The median Kurdistan all-cause and death-censored graft survivals, estimated from 16 patients, were 15 years, with the 95%CI ranging from 8.5 to 21.5 years.

The length of the post-transplant period significantly correlated with the severity of IF/TA (Rs = 0.770, *P* < 0.001) and TG (Rs = 0.464, *P* < 0.001). Logistic regression (Table 6) showed that IF/TA and positive C4d IF staining significantly predicted HD but that acute TCMR and ATI did not. DWFG was not significantly related to sex, donor source, IF/TA, TG, C4d staining, ATI, or acute TCMR. Ethnicity did not significantly influence HD or DWFG.

**Table 6:** Logistic regression models for hemodialysis graft failure and death with a functional graft (DWFG)

Variable	Odds ratio	*P*-value	95% CI
Hemodialysis initial model			
Age	1.014	0.25	0.991 to 1.037
Sex	0.484	0.04	0.242 to 0.967
Donor source	1.450	0.38	0.635 to 3.312
Ethnicity	0.936	0.81	0.546 to 1.604
Acute TCMR	0.413	0.08	0.152 to 1.123
ATI	0.701	0.47	0.270 to 1.821
C4d+	2.264	0.04	1.044 to 6.576
IF/TA	1.935	0.000	1.375 to 2.719
TG	1.038	0.83	0.731 to 1.476
Hemodialysis final model			
Sex	0.647	0.16	0.355 to 1.179
C4d+	3.427	0.01	1.325 to 8.865
IF/TA	2.957	0.000	1.734 to 2.927
DWFG initial model			
Age	1.018	0.24	0.988 to 1.049
Sex	0.582	0.25	0.233 to 1.453
Donor source	0.632	0.48	0.178 to 2.246
Acute TCMR	0.492	0.27	0.138 to 1.754
Ethnicity	0.475	0.07	0.211 to 1.070
ATI	1.339	0.60	0.453 to 3.962
C4d+	0.245	0.20	0.029 to 2.090
IF/TA	1.022	0.93	0.639 to 1.633
TG	1.112	0.68	0.672 to 1.843
DWFG final model			
C4d+	0.292	0.23	0.044 to 2.204
Ethnicity	0.591	0.18	0.267 to 1.277

*Abbreviations*: *TCMR* T cell mediated rejection, *ATI* acute  tubular injury, *IF/TA* interstitial fibrosis and tubular atrophy, *TG* transplant glomerulopathy

Survival is translated into graft failure and compared with 2000 and 2010 failure rates for living donor transplants in the 2017 United States Renal Data Service (USRDS) Annual Data Report [16]. The Kurdistan data resembles living-donor failure rates in the US in 2000 for all-cause and HD. It is notable that failure rates in both Kurdistan and the US in 2000 are moderately higher than the lowered rates achieved in the US by 2010 (Table 7). The Kurdistan death rates do not seem to have an important overall influence on graft loss. The clinical characteristics of the US and Kurdistan living donor recipients are noted below the table. The comparison shows that these are very different populations, with US recipients being older and much more frequently diabetic. A gender difference is present but is not striking.

**Table 7:** One- and five-year living donor transplant failure rates. Estimated current Iraqi Kurdistan rates (95% confidence intervals) are compared with reported United States (US) outcomes in 2000 and 2010 [16]

Site, year	One-year graft failure (%)			five-year graft failure (%)		
	All-cause	HD	DWFG	All-cause	HD	DWFG
Kurdistan, 2019	6.0 (4.4–7.6)	4.2 (2.8–5.6)	1.9 (1.1–2.7)	18.1 (13.8–22.4)	13.7 (9.8–17.6)	5.1 (2.7–7.4)
US, 2000	7.0	5.0	2.6	22.3	15.2	10.6
US, 2010	3.7	2.4	1.4	15.3	9.6	7.3

Characteristics of living donor transplant recipients: US patients [16–18]; male 63%, age 45 ± 16 years, 48% ≥ 50 years old, 14% ≥ 65 years old, 31% diabetic. Kudistan patients; male 77%, age 39 ± 13 years, 20% ≥ 50 years old, 1.8% ≥ 65 years old, 6.5% diabetic

*Abbreviations*: *HD* hemodialysis, *DWFG* death with functional graft

## Discussion

The clinical characteristics of our recipients are similar to patients in previous Iraqi studies, with transplanted kidneys all coming from living donors [4]. Despite the religious fatwa of 1986 (the Amman declaration) that permits the retrieval and transplantation of deceased donor organs, the numbers of such transplants in the Middle and Near East, while increasing, remain limited [2, 5, 14].

In the Kurdistan nephrology clinics, 77% of transplant recipients were male, fewer than 2% were over 65 years old, and just 6.3% were diabetic. It might appear that recipients were actively selected for favorable outcomes, but that is generally not the case. Medications, dialysis, and approximately 50% of transplant procedures are public expenditures. But finding a donor is a private expense, and compensated donors provide 82% of the transplants.

Recipient selection is largely determined by the financial circumstances of the family. Procuring a donor and private transplants are beyond the resources of most Iraqi families, and families with modest financial circumstances commonly limit transplantation to potentially “breadwinning” males. Older and sicker males and women are rarely financially independent, and only wealthier families choose transplantation for such patients. The need for second transplants is growing, but this adds to the already large burden on families, and few repeat procedures are performed. More preemptive transplants might be advantageous, but considerable patient denial is encountered as kidney failure becomes end-stage. Patients are typically dialysed for 2 to 4 months before they accept their condition as irreversible and make arrangements for a graft.

With this background, we show that the all-cause graft failure rates in Kurdistan are similar to the USRDS reported recipients of living donor kidneys in the year 2000 and are not greatly different than the US rates in 2010 [16]. Since 2010, the survival rates for US recipients of living donor kidneys have not appreciably changed, and 2010 seems to be a plateau that current findings at any location can be measured against [16]. The frequency of DWFG for Kurdistan patients is low at both 1 and 5 years after engraftment. The difference in all-cause failure between Kurdistan patients and US 2010 is related to a moderately higher frequency of return to hemodialysis in Kurdistan.

The US living donor kidney recipients are not comparable to the Kurdistan patients. The age and morbidity status of US living donor kidney recipients are similar to patients on waiting lists for deceased donor kidneys [16–18]. Nearly 50% of US living donor recipients are over 50 years old, 14% are 65 years of age or older, and more than 30% are diabetic [18]. The most frequent cause of DWFG in the US is cardiovascular disease [16, 17, 19]. With few diabetic and elderly patients, the number of Kurdistan transplant recipients at risk for cardiovascular-related DWFG is small. Sepsis and pneumonia were our most frequent causes of DWFG. The COVID-19 pandemic reached the Kurdish region in March of 2020, with a death rate among the COVID-19 positive general Kurdistan population of 3.3% [20]. Through the 2020 follow-up, COVID-19 caused four deaths among patients that were biopsied (0.9%). We concluded that COVID-19 did not influence our transplant survival and that the one-year DWFG rate of 1.9% was appropriate for the characteristics of the recipient population.

Kurdistan patients seem to have an increased frequency of acute TCMR. At 23% of all biopsies, acute TCMR was our most common cause of graft dysfunction. As in Western practices, acute TCMR was generally mild and well managed [21]. It was the attributed cause of just 8.5% of our graft loss, with half of this loss being DWFG or subsequent chronic changes resulting in IF/TA.

The frequency of acute TCMR in US practice is considered low, but it varies. Mehta et al. [22] surveyed the frequency of TCMR in US transplant centers. In the first year post-transplantation, 54% of centers had a biopsy frequency of TCMR of less than 10%, but 40% of centers reported 10–15, and 6% reported more than 15%. The Genome Canada [21] microarray studies suggest that biopsy and clinical criteria underestimate acute rejection and that 20% of patients may have TCMR around 100 days after engraftment.

The use of live donors should lessen the enhancing effect that prolonged cold ischemia time has on ATI and acute TCMR [23, 24]. Post-transplant ATI may be increased among Kurdistan patients, but like TCMR, ATI was usually mild and had little effect on the graft. While ATI and infarction resulted in 12.7% of graft loss, infarction complicating vascular anastomoses caused nearly half of that loss. Half of the failures associated with ATI were DWFG, suggesting that something more severe than temporary operative ischemia compromised patient outcomes. The complexity of acute post-transplant dysfunction is emphasized in the Genome Canada results [21]. The authors found that TCMR had little effect on graft survival. They also observed that the cause of progressive transplant injury could not be identified in many cases [21].

Our estimated 10-year death-censored survival rate of 66.2% is encouraging, but prolonged survival comes with additional contributions to graft loss. We see the shift toward chronic and antibody-mediated transplant disorders that has taken place in developed countries [6, 7]. Parajuli et al. [8], at the University of Wisconsin transplant service, retrospectively examined biopsies from 329 patients that developed graft failure over the years, 2006–2016. They had a population of highly sensitized recipients, and active AMR caused 32% of graft failures. TG at 17% and IF/TA at 13% followed active AMR.

Naesens et al. [25] examined the transplant biopsies of 1197 patients from the transplant service of the University Hospital of Leuven, Belgium. The donors were 99% deceased, and 33% of procedures were repeated transplants. After the second year post-transplantation, IF/TA, TG, and late TCMR were the most common causes of graft failure, and the severity of IF/TA with any diagnosis correlated strongly with graft loss. Like Parajuli et al. [8], the Leuven study found an increased frequency of AMR, and AMR significantly lowered graft survival [25].

Our patients have different characteristics than those of the Leuven or Wisconsin transplant services. Yet, we see the same influence of AMR, TG, and IF/TA on graft survival. De novo active AMR was associated with more than 8% of our graft dysfunction, and after 1 year, chronic active AMR was 56% as frequent as TG. This proportion of chronic active AMR to TG is similar to the 53% found 1 year after engraftment in Leuven, with the ratio possibly reflecting the comparative risk of TG as a non-complement binding DSA-mediated disease [25, 26].

Because TG and chronic active AMR are frequent causes of progressive late graft dysfunction, their management is an important concern for nephrologists [9, 27, 28]. While the reduction in proteinuria by angiotensin II blockade is the usual treatment for TG, the recognition that TG may be an alloimmune disease suggests that immunosuppression, including the use of rituximab, might be effective [12, 28, 29]. The treatment of chronic active AMR can similarly be directed at the antibody disease, with options that include steroid pulse therapy, rituximab, intravenous immunoglobulin, and bortezomib [30]. The diagnoses and the treatment of TG and chronic active AMR are likely to be refined as sizable case series and clinical trials are reported [30–32].

The diagnosis of IF/TA presents uncertainties equal to TG. IF/TA is the late stage of different pathologies, and the cause is by definition obscure [33]. In two of our cases, IF/TA was preceded by ATI, a relationship recently recognized by Gosset et al. [34]. ATI carries an increased risk of later CKD, and we have documented the progression of ATI to chronicity in Iraqi community patients and now in transplants [35–37]. There is no currently recommended treatment for idiopathic IF/TA, but recognizing and intervening in causal events might moderate progression in some cases.

The Kurdistan patients consist of a majority of new transplants. In 2019, 62.2% received a graft, with only 3.7% being second transplants. In the foreseeable future, dialysis in Iraq, as in most developing countries, is likely to remain limited, and the demand for transplants will increase. As this study shows, there is a substantial population of long-term survivors growing in parallel to the new transplants. Chronic transplant disorders are currently the most frequent cause of graft loss among our patients and are creating a population of secondary ESRD patients. As the treatment of chronic graft disorders is better understood, the progression to ESRD may be delayed, but eventually, a second transplant or maintenance dialysis will be needed.

Unless health ministries plan for adequate dialysis facilities, repeat transplantation is likely to remain the principal option for further long-term survival. For properly managed patients, the outcomes for second transplants can be as good as those for primary transplants [38]. This success, of course, requires donor-recipient compatibility [39, 40]. Some Middle-East centers have the facilities and, even more importantly, the knowledge to optimize donor selection [40]. This capacity is poorly developed in Iraq, and the management of repeat transplants will be challenging.

The ability to collect patient data is a major limitation to this study. Renal biopsies and records are kept in the Sulaimania pathology laboratory with patient-specific codes, and the information on renal biopsies is readily collected and verified. Otherwise, centralized record-keeping does not exist in Iraqi or most low-resource countries [41]. Determining the number of patients depends upon examining handwritten daily or weekly clinic records or, in the case of private clinics, Excel™ or Access™ files. These records contain names but no identifying codes, and the chance of duplicating or not identifying patients is high. We have encountered this issue with studies on breast cancer and have suggested that the patient counting error is approximately 10–15% [42]. A 10% undercount would increase the one-year failure rate to 6.4 (95%CI 4.7–8.1), a slightly worse but trivial difference from what is reported in Table 7. An overcount would bias outcomes in the opposite direction and make survival appear better than it might actually be.

Obtaining patient follow-up is similarly laborious. Within the first year, kidney transplant patients have monthly to bi-monthly appointments. From 1 to 2 years, patients are seen at four to six-month intervals. Except for hospitalizations, patients rarely miss these appointments, and short-term status is readily determined. After 2 years, outcomes become more difficult to establish and usually depend upon mobile telephone calls to patients or their families. Nevertheless, our follow-up failure rate of 4.5% is low and should be acceptable by any standard.

The short-term estimates can be considered reasonably accurate, but data becomes increasing increasingly poor with the length of time post-transplantation. The wide confidence intervals for 10-year survival reflect this uncertainty, and the 15-year determination for median survival is highly tenuous.

## Conclusions

In this Kurdistan transplant population, one- and five-year all-cause and HD graft survivals match those for recipients of living donor kidneys reported by the USRDS for the year 2000 and are not greatly different than for 2010 and afterward. Few elderly or diabetic patients are transplanted in our region, and rates of DWFG are low. We show that the most frequent causes of graft failure are TG, IF/TA, and chronic active AMR occurring at a median of 3.1 years post-transplantation. Nearly 15% of these patients have acquired DSA. These chronic disorders are creating a population with recurring ESRD, and many are likely to request second transplants. For patients with DSA, providing donor-recipient compatibility will challenge our technical abilities.

## Data Availability

Compiled data and calculations are stored in Excel files in the Shorsh University Hospital Pathology Department and will be made available upon request to the corresponding author.
